# Patterns of expression of VEGFR2, PDGFRs and c-Kit in pediatric patients with high grade non-rhabdomyosarcoma soft tissue sarcoma

**DOI:** 10.3389/fonc.2024.1480773

**Published:** 2024-10-29

**Authors:** Mona M. Mohammed, Hanafy A. Hafez, Enas M. Elnadi, Asmaa I. Salama, Abd Elaziz Saad Abd Elaziz, Gehad T. Ahmed, Madeeha A. ELwakeel, Mohamed K. Kamal, Mark W. Kieran, Alaa M. Elhaddad

**Affiliations:** ^1^ Department of Pediatric Oncology, Children Cancer Hospital (57357), Cairo, Egypt; ^2^ Department of Pediatric Oncology, National Cancer Institute, Cairo University, Cairo, Egypt; ^3^ Department of Pediatric Oncology, Beni-Swief University, Beni-swief, Egypt; ^4^ Department of Pathology, Children Cancer Hospital (57357), Cairo, Egypt; ^5^ Department of Pathology, National Cancer Institute, Cairo University, Cairo, Egypt; ^6^ Department of Surgical Oncology, Children Cancer Hospital (57357), Cairo, Egypt; ^7^ Department of General Surgery, Faculty of Medicine, Helwan University, Cairo, Egypt; ^8^ Department of Radio-Diagnosis, Children Cancer Hospital (57357), Cairo, Egypt; ^9^ Department of Radio-Diagnosis, National Cancer Institute, Cairo University, Cairo, Egypt; ^10^ Department of Clinical Research, Children Cancer Hospital (57357), Cairo, Egypt

**Keywords:** VEGFRs, PDGFRs, c-kit, pediatric, sarcoma, immunohistochemistry

## Abstract

**Introduction:**

Activated vascular endothelial growth factor receptors (VEGFRs), platelet-derived growth factor receptors (PDGFRs) and c-Kit have been shown to be involved in the growth, invasion and metastasis of non-rhabdomyosarcoma soft tissue sarcoma tumor (NRSTS) with promising results for targeted therapy. Our aim was to assess the expression of these markers among different histological types and correlate with outcomes.

**Material and methods:**

This retrospective study included pediatric patients aged ≤ 18 years diagnosed with high-grade NRSTS who were treated at Children Cancer Hospital Egypt 57357 as per the COG NRSTS protocol (ARST0332). Expression of VEGFR2, PDGFRs (α and β) and c-Kit in tumor tissue was assessed by immunohistochemistry and correlated with clinical outcome.

**Results:**

Of 113 patients, 96 were eligible for the analysis with a median age of 11 years. Overall, 32.3% demonstrated high expression of PDGFRα, 17.7% for PDGFRβ, 19.8% for VEGFR2 and 8.3% exhibited positive expression for c-kit on the tumor cells. Most cases of synovial sarcoma (45.8%) and 43.7% of patients with undifferentiated sarcoma exhibited high expression of PDGFRα while 41.6% of MPNST showed high expression to PDGFRβ. The 5-year overall survival (OS), event free survival and relapse free survival (RFS) for the whole cohort were 59%, 54% and 60% respectively. In univariate analyses, only PDGFRα had a negative prognostic impact on relapse free survival (RFS) (*p*=0.03). In multivariate analyses, VEGFR2 was found to have a negative prognostic impact for OS (*p* = 0.02).

**Conclusion:**

Our findings indicated that tyrosine kinase receptors are upregulated in NRSTS and exhibited a distinct expression pattern within various subgroups. High expression of VEGFR2 and PDGFRα significantly correlated with reduced survival and may guide targeted therapy approaches for this poor prognosis group of patients.

## 1 Introduction

Non-Rhabdomyosarcoma soft tissue sarcomas (NRSTS) are relatively uncommon cancers, accounting for approximately 3.5% of all childhood tumors. They represent a challenge for treatment due to their diverse biology, requiring treatment that has to be tailored to the specific tissue type (1). Histologic grade is one of the most important prognostic factors in the outcome of soft tissue sarcoma (STS) as patients presenting with high-grade lesions are much more likely to develop metastatic disease resulting in poor survival (2). The optimal therapeutic management requires a multi-disciplinary approach adapted to each patient and can include surgical resection alone or in combinations of radiotherapy with or without chemotherapy. Neoadjuvant chemotherapy and radiotherapy have been widely used for patients with advanced soft tissue sarcomas (considered deep seated tumors >5cm, those that are unresectable or those with metastatic disease), with radiological response rate of only 35-40% (3, 4). The role of adjuvant chemotherapy in high-grade NRSTS is still controversial due to the small effect on outcome and the variability of response among different histological subtypes (5). Despite intensive multimodality therapy, patients with high-risk metastatic disease have 5-year event-free survival (EFS) of only 21·2% and overall survival (OS) of 35·5%. Given the limited efficacy of chemotherapy in NRSTS and the poor outcomes of a significant fraction of the patient population, and recognizing that further intensification of cytotoxic chemotherapy is not feasible due to severe toxicity, novel therapeutic approaches are needed (6).

Solid tumors are dependent on the vascular system for growth, invasion and metastasis. Tumor angiogenesis has been shown to support solid tumors mainly by supplying oxygen, glucose and cytokines as well as providing the means to the formation of metastases. A number of growth factors, proteases and cytokines have been reported to have pro-angiogenic effects and to induce tumor angiogenesis (7). Human sarcomas express a number of proangiogenic factors that may represent potential therapeutic targets. Certain tyrosine kinases have been found to be expressed in a range of NRSTS subtypes. The vascular endothelial growth factor receptors (VEGFRs, 1-3), platelet-derived growth factor receptors (PDGFRs, α and β), and c-Kit pathways are among the most commonly dysregulated in soft tissue sarcomas (8). The vascular endothelial growth factors (VEGFs) and their receptors play a distinct role in the growth of tumor vasculature through regulation of endothelial cell proliferation and sprouting which drives angiogenesis and lymphangiogenesis. Signaling through VEGFR2 is considered the major angiogenic pathway, prompting endothelial cells (ECs) to proliferate and form tubes (9). Some studies had correlated high expression of VEGFR2 with decreased survival of various soft tissue tumors (10, 11). The PDGFRs are structurally related tyrosine kinase receptors consisting of either α or β chains and function to activate pericytes which support new microvasculature and play a major role in angiogenesis and regulation of tumor stroma (10). Additionally, PDGFRs (α and β) play a key role in activating the PI3K/Akt/mTOR (phosphatidylinositol 3-phosphate kinase/protein kinase B (PKB)/mammalian Target of Rapamycin) pathway in sarcomas which in turn, initiates a cascade of downstream signaling that promotes cell proliferation and survival, making this pathway a critical target for therapeutic development in sarcomas like mTOR inhibitors (12). Activated VEGFRs and PDGFRs work together to guide the microvasculature into tumor lesions, and are reported to be involved in tumor growth, invasion and metastasis (13). The proto-oncogene c-Kit (CD117) encodes a transmembrane tyrosine kinase receptor and its expression has been detected in a variety of different tumor entities such as gastrointestinal stromal tumors (GIST), malignant melanoma, breast and lung cancer, sarcoma and mastocytosis (14). Activating mutations in c-Kit play a pivotal role in the pathogenesis of the majority (90-95%) of GIST. The standard diagnostic workup of GIST tumors now includes assessment of c-Kit abnormalities and a number of c-kit inhibitors, such as imatinib, have demonstrated significant activity in tumors with these mutations (15).

Several agents targeting VEGFRs, PDGFRs and c-Kit activity are now being introduced for the treatment of sarcomas with promising results like imatinib, sorafenib, sunitinib, anlotinib and pazopanib (16). The multi-targeted tyrosine kinase inhibitor pazopanib, is a potent inhibitor of these pathways, and has shown potential for synergistic interaction with conventional cytotoxic chemotherapy in preclinical studies, suggesting that these drug combinations might overcome chemo-resistance (17, 18). Pazopanib improved outcomes in adults with advanced soft tissue sarcomas and was approved for single-agent use by the US Food and Drug Administration in adults with advanced soft tissue sarcomas based on results from the phase 3 randomized PALETTE study. In this study, patients treated with pazopanib experienced significantly better progression-free survival compared to those given a placebo (median 4.6 months versus 1.6 months, respectively; hazard ratio = 0.31; P < 0.0001). In addition, Cox models revealed a significant benefit for progression-free survival across all histological subtypes receiving pazopanib (19). The COG ARST1321 study added pazopanib to neoadjuvant chemo-radiotherapy, which resulted in increased rates of pathological near complete response, suggesting that this is a highly active combination in children as well as adults with advanced soft tissue sarcomas (20). Although pathological response is still not considered a prognostic factor, and long-term data is lacking, it remains crucial to determine whether a greater pathological response rate will translate into improved local control and survival in NRSTS.

Additionally, recent advancements have identified new therapeutic targets in the treatment of STS, with promising new agents currently under clinical investigation. Monoclonal antibodies such as ramucirumab which specifically VEGFR2 (21) and olaratumab, targets PDGFR α (22) are among these agents. Furthermore, nintedanib, a potent triple angiokinase inhibitor targets PDGFR, VEGFR, and FGFR pathways, has demonstrated significant anti-proliferative effects *in vitro* (23). Despite the recent emergence of these attractive molecular targets, there is limited data available regarding the association between these targets and pediatric NRSTS. To address this gap, this study was designed to assess the expression status of VEGFR2, PDGFRs (α and β) and c-Kit in relation to different histological subtypes of high-grade NRSTS and to estimate the impact of overexpression on treatment outcome to traditional therapies.

## 2 Patients and methodology

### 2.1 Patient selection

A total of 113 pediatric patients ≤18 years old with newly diagnosed high-grade NRSTS from January 2013 to January 2020 who were treated at Children Cancer Hospital 57357-Egypt (CCHE-57357) as per the COG NRSTS protocol ARST0332 were considered for inclusion. Of these 113, 96 patients met the eligibility criteria for the study. Of the 17 patients who were excluded, 15 had insufficient tumor material for analysis, 1 patient died before starting treatment and one patient refused treatment. Demographic and clinical data (histological subtype, tumor site, tumor size and stage) were collected from the medical records. The minimum follow up period was 2 years from the end of treatment. Primary tumor tissue was obtained at diagnosis and histologically subtyped according to the World Health Organization guidelines (WHO 2020) and graded according to the French Fédération Nationale des centres de Lutte Contre le Cancer (FNCLCC) system (24, 25). The institutional review board approved this retrospective study.

### 2.2 Immunohistochemistry

Formalin-fixed and paraffin-embedded primary tumor specimens (FFPE) were obtained from the archive of the Pathology Departments and four um-thick sections were prepared using a microtome (Leica Biosystems) and mounted on positive charged slides. The antibodies used were as follows: vascular endothelial growth factor receptor2 (VEGFR2, 1:50, rabbit monoclonal, D5B1, Cell Signaling), Platelet derived growth factor receptor alpha (PDGFR α, 1:100, rabbit monoclonal, D13C6, Cell Signaling), Platelet derived growth factor receptor beta (PDGFR β, 1:100, rabbit monoclonal, C82A3, Cell Signaling), C-Kit (Ready to use, rabbit monoclonal CD117, YR145, Cell Marque). The cellular localization for all antibodies was cytoplasmic reaction (see **Supplementary Figures 1**, **2**).

### 2.3 Immunohistochemistry scoring method

For VEGFR2, PDGFRα and PDGFRβ biomarkers, the immune reactive scoring (IRS) method as previously published was used to assess the degree of cytoplasmic intensity (negative, weak, intermediate or strong) and percentage of tumor cells expressing the reaction (26); see **Supplementary Table 1**. Tumor tissues with IRS negative or weak scores were considered to have low expression while those with moderate or strong IRS scores were considered to have high expression. Assessment of c-Kit expression used a minimum cut off of 10% as previously published (27).

### 2.4 Statistical analysis

Statistical analyses were performed by R software for statistical computing version 4.2.2. Descriptive statistics were calculated and shown for expression of all biomarkers. The Chi-square test and Fishers Exact test were used to examine the association between marker expression and various clinico-pathological parameters. Univariate and multivariate analyses were performed using Cox’s proportional hazard model. In multivariate analyses, factors included site, stage, VEGFR2, PDGFRs (α and β) and c-Kit expression. Overall survival (OS) was calculated from the date of diagnosis until the date of death or last contact in the clinic. Event free survival (EFS) was calculated from the date of diagnosis until the first event, including progression, relapse or death. Relapse free survival (RFS) was calculated from treatment to the time of relapse. The log rank test was used to detect differences between survival curves for stratified variables. Significance was presented by p value if less than 0.05.

## 3 Results

### 3.1 Patient’s characteristics

The median age of the analysis cohort (n=96) was 11 years (range 0.05-17 years), with a male to female ratio of 0.8:1. More than 50% of the tumors were located in the trunk 61.5% (n=59) while 38.5% of the tumor originated in the extremities (n=37). Of the 96 patients, tumor size was >5 cm in 71.9% (n=69) and ≤5 cm in 28.1% (n=27). The most common represented histological subtypes were synovial sarcoma (n=24), undifferentiated sarcoma (n=16), malignant peripheral nerve sheath tumor (MPNST) (n=12), epithelioid sarcoma (9) and sarcoma with BCOR genetic alterations (n=7). In accordance with the COG protocol, surgery alone (Arm A) was performed in seven patients (7.3%), surgery followed by radiotherapy (Arm B) in 17.7% (n=17), and surgery followed by adjuvant chemotherapy and radiotherapy (Arm C) in 19.8% (n=19). Fifty-three patients (55.2%) had inoperable tumors, and were treated with neoadjuvant chemotherapy, then surgery followed by adjuvant chemotherapy and radiotherapy (Arm D).

Among the 96 patients, high expression of PDGFRα was demonstrated in 32.3% (n=31) of patients′ tissue samples, VEGFR2 in 19.8% (n=19) and PDGFRβ in 17.7% (n=17) of cases. Positive expression of c-kit was identified in 8.3% (n=8). Specifically, 45.8% (n=11) of synovial sarcoma cases and 43.7% (n=7) of patients with undifferentiated sarcoma showed high expression of PDGFRα. Furthermore 41.7% (n=5) of patients with MPNST exhibited high expression of PDGFRβ. Collectively, expression of one or more of the target markers was documented in 58.4% of synovial sarcoma, 56.3% of undifferentiated sarcoma and 58.3% of MPNST. Additionally, among the seven cases of sarcoma with BCOR genetic alterations, six patients (85.7%) exhibited high expression of various markers (four for c-kit, six for PDGFRα, and two for VEGFR2). Five of the patients with BCOR genetic alterations presented with advanced (III and IV) stage and were treated with neo-adjuvant chemo-radiotherapy with no responses observed. A detailed descriptive analysis of marker expression across different histological subtypes is shown in **Table 1**.

**Table 1:** Descriptive data for tumor expression of c-Kit, VEGFR2, PDGFRα, PDGFRβ between different histological subtypes in the 96 pediatric patients with NRSTS.

Histological subtypes (no)	c-Kit		VEGFR2		PDGFRα		PDGFRβ	
	Negative **No. (%)**	Positive **No. (%)**	**Low** **No. (%)**	High **No. (%)**	**Low** **No. (%)**	High **No. (%)**	**Low** **No. (%)**	High **No. (%)**
**Synovial Sarcoma** (24)	23 (95.8)	1 (4.2)	20 (83.3)	4 (16.7)	13 (54.2)	11 (45.8)	22 (91.7)	2 (8.3)
**Undifferentiated Sarcoma** (16)	12 (75)	4 (25)	12 (75)	4 (25)	9 (56.3)	7 (43.7)	12 (75)	4 (25)
**MPNST** (12)	12 (92.3)	–	10 (83.3)	2 (16.7)	11 (91.7)	1 (8.3)	7 (58.3)	5 (41.7)
**Epithelioid sarcoma** (9)	9 (100)	–	8 (88.9)	1 (11.1)	8 (88.9)	1 (11.1)	6 (66.7)	3 (33.3)
**Alveolar soft part sarcoma**	6 (100)	–	6 (100)	–	6 (100)	–	6 (100)	–
**Sarcoma with BCOR genetic alterations** (7)	3 (42.9)	4 (57.1)	5 (72.5)	2 (28.5)	1 (14.3)	6 (85.7)	7 (100)	–
**CIC rearranged sarcoma** (4)	4 (100)	–	3 (75)	1 (25)	3 (75)	1 (25)	4 (100)	–
**Extraskeletal Chondrosarcoma** (6)	5 (83.3)	1 (16.7)	5 (83.3)	1 (16.7)	5 (83.3)	1 (16.7)	6 (100)	–
**Clear cell sarcoma** (5)	5 (100)	–	4 (80)	1 (20)	5 (100)	–	5 (100)	–
Others* (7)	6 (85.7)	1 (14.3)	4 (57.1)	3 (42.9)	4 (57.1)	3 (42.9)	4 (57.1)	3 (42.9)

VEGFR2, vascular endothelial growth factor receptors; PDGFR, platelet-derived growth factor receptors; MPNST, malignant peripheral nerve sheath tumor.* Others: 1 Leiomyosarcoma, 1 angiosarcoma, 2 Embryonal Sarcoma, 2 Myxoid Liposarcoma, 1 Myxofibrosarcoma.

Among the patients, 53 presented with advanced unresectable disease, (34 with stage III and 19 with stage IV). These patients were treated with neoadjuvant chemotherapy resulting in partial response in 35.8% (n=19) patients, stable disease in 43.5% (n=23), disease progression in 13.2% (n=7) during chemotherapy while four patients (7.5%) succumbed to their disease before achieving local control. When analyzing markers expression in this patient group, high expression of PDGFRα, VEGFR2, PDGFRβ and c-kit was exhibited in 37.7% (n=20), 22.6% (n=12), 15% (n=8) and 11.3% (n=6) of tumors, respectively. A correlation with markers expression in patients treated with chemotherapy was not significant, likely due to small number of patients who showed response to chemotherapy.

Correlation between the expression of target markers and the patients’ clinic-pathological variables including site (extremities vs. trunk), size (≤5cm vs. >5 cm) and stage (stage I+II vs. III vs. IV) of disease revealed no significant differences, summarized in **Table 2**.

**Table 2:** Descriptive and statistical analysis for the expression of angiogenic markers based on clinic-pathological factors for the 96 pediatric patients with NRSTS.

	Patient number (%)	c-Kit		P value	VEGFR2		P value	PDGFRα		P value	PDGFRβ		P value
**Clinico-pathological variables** **no (%)**	Total n=96 (100%)	Negative No (%)	Positive No (%)		Low No (%)	High No (%)		Low No (%)	High No (%)		Low No (%)	High No (%)	
**Site** Trunk Extremities	59 (61.5) 37 (38.5)	53 (89.8) 35 (94.5)	6 (10.2) 2 (5.5)	0.48	48 (81.4) 29 (78.4)	11 (18.6) 8 (21.6)	0.79	41 (69.5) 24 (64.9)	18 (30.5) 13 (35.1)	0.66	49 (83) 30 (81)	10 (17) 7 (19)	0.79
**Size** ≤5 cm >5 cm	27 (28.1) 69 (71.9	24 (88.9) 64 (92.8)	3(11.1) 5 (7.2)	0.68	20 (74) 57 (82.6)	7 (26) 12 (17.4)	0.4	19 (70.4) 46 (66.7)	8 (29.6) 23 (33.3)	0.81	22 (81.5) 57 (82.6)	5 (18.5) 12 (17.4)	1
**TNM stage** I+II III IV	23 (24) 54 (56.2) 19 (19.8)	21 (91.3) 50 (92.6) 17 (89.5)	2 (8.7) 4 (17.4) 2 (10.5)	1	17 (74) 47 (87) 13 (68.4)	6 (26) 7 (13) 6 (31.6)	0.38	15 (65.2) 36 (66.7) 14 (73.7)	8 (34.8) 18 (33.3) 5 (26.3)	0.8	18 (78.3) 41 (75.9) 18 (94.7)	5 (21.7) 11 (24.1) 1 (5.3)	0.54

VEGFR2, vascular endothelial growth factor receptors; PDGFR, platelet-derived growth factor receptors; TNM, Tumor size; Nodal involvement, Metastasis.

### 3.2 Survival outcome

**Figure 1:**
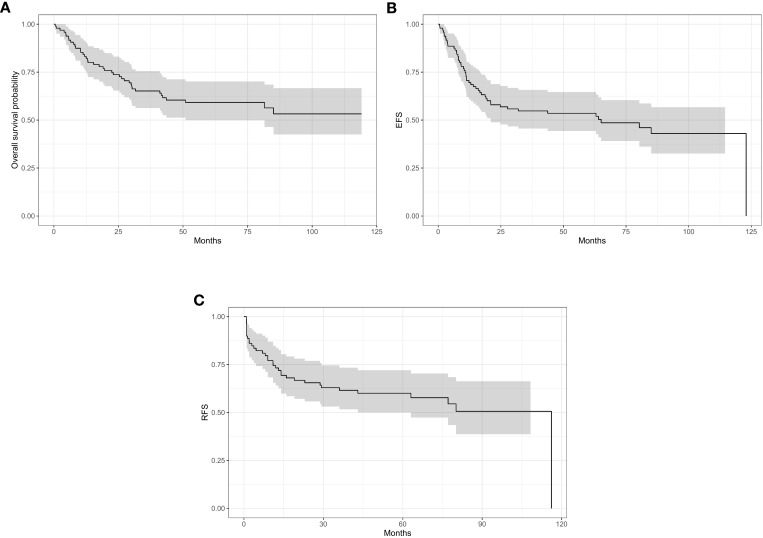
5-year survivor for the whole cohort: **(A)** overall survivor (OS) 59%. **(B)** event free survivor (EFS) 54%. **(C)** relapse free survivor (RFS) 60%.

The median follow-up period was 71.06 months (range 52.86- 90.15) with a 5-year OS, EFS and RFS for the whole patient cohort of 59%, 54% and 60% respectively (95% CI) (**Figures 1A–C**). At the time of data analysis, 54 patients were alive, 39 patients had disease progression and died while only three of the 96 patients were lost to follow-up.

The results suggest that tumors located in the trunk have a negative prognostic impact on OS and EFS compared to those in the extremities. Specifically, OS rates were 48% for trunk tumors versus (vs) 77% for extremity tumors (P<0.01), and EFS rates were 40% for trunk tumors vs 75% for extremity tumors (P<0.01). Regarding RFS, the rates were 49% for trunk tumors and 76% for extremity tumors, with a p value of 0.05, almost reaching statistical significance. For tumor size, larger tumors> 5 cm had poorer outcome versus tumors ≤5 cm in size for OS, EFS and RFS with rates of 48% vs 89% (P<0.01), 43% vs 80% (P<0.01), and 49% vs 82% (P=0.02) respectively. Additionally, advanced tumor stage was associated with inferior survival outcome for stages I+II vs III vs IV, where OS 91% vs 64% vs 11% (P<0.01), EFS 81% vs 57% vs 11% (P<0.01), RFS 80% vs 59% vs 20% (P<0.01) respectively (**Table 3**). In univariate analysis, the impact of angiogenic markers on survival outcome was only observed in tumors with high expression of PDGFRα in which a significant reduction in RFS was observed versus patients with low expression (43% vs 70%, CI 95%, *P=0.03*). None of the other markers had an impact on OS or EFS. The impact of clinico-pathological factors and target markers on survival are summarized in **Table 3**.

**Table 3:** Prognostic relevance of clinicopathological variables and angiogenic markers for OS, EFS, RFS in 96 pediatric patients with high-grade NRSTS.

Characters:	Patient number (%)	5-year OS (%)	P value	5-year EFS (%)	P value	5-year RFS (%)	P value
**Site** Trunk Extremities	59 (61.5) 37 (38.5)	48 77	0.009	40 75	0.004	49 76	0.05
**Size** ≤5 cm >5 cm	27 (28.1) 69 (71.9	89 48	<0.001	80 43	<0.001	82 49	0.02
**TNM stage** I+II III IV	23 (24) 54 (56.2) 19 (19.8)	91 64 11	<0.001	81 57 11	<0.001	80 59 20	<0.001
Markers expression							
**c-Kit** negative positive	88 (91.7) 8 (8.3)	58 73	0.4	53 56	0.5	60 55	0.9
**VEGFR2** Low High	77 (80.2) 19 (19.8)	62 49	0.3	54 51	0.7	60 62	0.8
**PDGFRα** Low High	65 (67.7) 31 (32.3)	61 56	0.9	56 47	0.4	70 43	0.03
**PDGFRβ** Low High	79 (82.3) 17 (17.7)	61 49	0.5	56 44	0.7	63 47	0.3

OS, overall survivor; EFS, event free survivor; RFS, relapse free survival; VEGFR2, vascular endothelial growth factor receptors; PDGFR, platelet-derived growth factor receptors; TNM, Tumor size, Nodal involvement, Metastasis.

### 3.3 Multivariate analysis

In multivariate analyses of OS and EFS including site, stage, VEGFR2, PDGFRs (α and β) and c-Kit, tumor stage III-IV (*P<0.01*) and high expression of VEGFR2 (*p=0.02)*, were significant independent prognostic indicators for OS. The other markers including c-Kit (*p=0.13*), PDGFRα (*p=0.9*), PDGFRβ (*p=0.88*) and tumors in the trunk site (P=0.05*)* failed to demonstrate a prognostic impact. Regarding EFS, only tumor site (*p=0.015*) reached statistical significance while stage III-IV (p=0.05), c-Kit (*p=0.16*), VEGFR2 (*0.29*), PDGFRα (*p=0.0.25*) or PDGFRβ expression (*p=0.8*) failed to reach statistical significance. Results of the multivariate analyses for OS and EFS are presented in **Tables 4** and **5** respectively.

**Table 4:** Multivariate cox regression for OS including variables site, stage, c-Kit, VEGFR2, PDGFRα, PDGFRβ.

Factor	Hazard ratio	Confidence interval (95%)	P value
**Site** Extremities trunk	2	0.98-4.2	0.05
**TNM Stage** I-II III-IV	7.2	1.68-31.1	0.008
**VEGFR2** Low High	2.6	1.15-5.8	0.02
**PDGFRα** Low high	1	0.52-2.1	0.9
**PDGFRβ** Low high	1.1	0.47-2.4	0.8

OS, overall survival; VEGFR2, vascular endothelial growth factor receptors; PDGFR, platelet-derived growth factor receptors; TNM, Tumor size, Nodal involvement, Metastasis.

**Table 5:** Multivariate cox regression for EFS including variables site, stage, c-Kit, VEGFR2, PDGFRα, PDGFRβ.

Factor	Hazard ratio	Confidence interval (95%)	P value
**Site** Extremities trunk	2.3	1.18-4.6	0.015
**TNM Stage** I-II III-IV	2.4	1.00-6.0	0.051
**VEGFR2** Low High	1.5	0.7-3.3	0.2
**PDGFRα** Low high	1.42	0.7-2.6	0.8
**PDGFRβ** Low high	0.95	0.4-2.0	0.8

EFS, event free survival; VEGFR2, vascular endothelial growth factor receptors; PDGFR, platelet-derived growth factor receptors; TNM, Tumor size, Nodal involvement, Metastasis.

## 4 Discussion

Angiogenesis is important for tumor growth and metastasis. Extensive research on angiogenesis in patents with STS has discovered a series of pro-angiogenic factors that can have direct or indirect influence on tumor angiogenesis (7). Among these, the VEGF receptors, platelet-derived growth factor receptors, and c-Kit are the most commonly dysregulated pathways in STS (28). Effective novel targeted therapies that disrupt the crosstalk between stroma and tumor cells are a promising strategy for cancer treatment (29). Previous retrospective studies in STS have consistently demonstrated a significant correlation between high expression of angiogenic markers and higher-grade sarcomas. However, the majority of these studies primarily included NRSTS of all grades (G1-G3) and focused on adult populations, with limited numbers of pediatric cases owing to the rarity of the disease in this age group (10, 30, 31). Considering that NRSTS are a very heterogeneous group of diseases and the expression of the markers are variable among different histological subtypes, we analyzed the prevalence of VEGFR2, PDGFRs (α and β) and c-Kit in a well-characterized group of pediatric patients with high-grade NRSTS, aiming to identify biomarkers in distinct subgroups that might benefit from a more individualized therapy approach. In 2015, Kampmann et al. investigated the expression of VEGFRs (1–3) and PDGFRs (α and β) in 275 adult patients with grade 2 and 3 soft tissue sarcoma using immunohistochemistry. Their findings demonstrated that high expression of VEGFR2 (p = 0.032) was an independent poor predictor of long-term survival. By contrast, this effect was not observed for PDGFRs α or β expression (10). Moreover, Kilvaer and coworkers published a study on 181 patients (12 patients were younger than 20 years old) with STS. They demonstrated the influence of VEGFRs on recurrence-free survival, metastasis free survival and disease-specific survival on a variety of subgroups defined by tumor site (31).

In addition to these studies, further investigations have explored these pathways across various histological subtypes, including malignant peripheral nerve sheath tumors (MPNST) and synovial sarcoma. Notably, a study by Perrone et al. demonstrated upstream activation of PDGFR (α and β) and EGFR (endothelial growth factor receptor), alongside downstream RTK signaling activation in MPNST. These findings suggest that combined inhibition of RTK and mTOR pathways could be an effective therapeutic approach for MPNST (32).

Furthermore, a previously published study involving 255 pediatric and adult STS patients examined the differential expression of PDGF ligands and receptors across sarcoma subtypes. The results revealed a significant correlation between PDGF α expression and the risk of metastatic relapse (P = 0.006), further indicating that the expression levels of these ligands and receptors are associated with sarcoma patient outcomes. These findings highlight their potential role as biomarkers for predicting the efficacy of PDGFRα-targeted therapies within this heterogeneous disease group (33).

In line with the findings of the previous studies, we identified VEGFR2 high expression exhibited a negative prognostic impact on OS (P=0.02), while high PDGFRα expression had a negative prognostic impact on RFS (P=0.03) in pediatric patients. Overall, our results emphasize the significance of both VEGFR2 and PDGFRα as prognostic markers of survival, while highlighting the need for additional studies to clarify the role of PDGFRβ in this context. Additionally, for c-Kit, immunostaining is a well-established diagnostic tool for GISTs and Imatinib has been shown to be a selective inhibitor of the tyrosine kinase activity of c-Kit (15). There have been occasional reports of its expression in a limited number of soft tissue tumors beyond GISTs (34, 35) and this is supported by the results presented in this study.

In our cohort, we observed IHC staining for c-Kit in a notably restricted number of soft tissue tumors, while no instance of clear cell sarcoma, MPNST, epithelioid sarcoma and alveolar soft part sarcoma exhibited c-Kit positivity. The significance of c-Kit positive staining on patients’ outcome is unclear as this did not impact outcome in the limited patient population in this series. This is in concordance with the finding of Hornick and Fletcher who conducted a study of 365 soft tissue sarcoma of different histological subtypes assessing c-Kit expression using IHC staining. Their results indicated that only 22 cases were c-kit positive and none were identified in patients with clear cell sarcoma, MPNST, epithelioid sarcoma or alveolar soft part sarcoma (36). Potti et al. studied the expression of c-Kit in 90 patients with STS with a mean age at diagnosis of 56.9 years. Only 20/90 patients showed overexpression of c-Kit and there was no impact on outcome (34). Along with the studies mentioned above, our data shows the limited utility of c-Kit immunostaining as a prognostic marker in certain subtypes of STS, emphasizing the importance of comprehensive diagnostic approaches tailored to the specific histological characteristics of each tumor subtype. Whether targeting of c-kit can add to the activity of existing therapy in this small subgroup of patients remains to be determined.

When evaluating the expression of the markers among different histological subtypes, distinct patterns emerged. For instance, synovial sarcoma and undifferentiated sarcoma showed high expression for one or more of the markers with predominant high expression of PDGFRα in 58.4% and 56.2% of cases respectively. In cases of MPNST, 58.3% of case showed predominant high expression of PDGFRβ. Additionally, more than 55% of patients with epithelioid sarcoma exhibited high marker expression with PDGFRβ. Furthermore, we investigated seven cases of sarcoma with BCOR genetic alterations and four patients with CIC-rearranged sarcoma, which are new subsets of rare sarcomas know with poor prognosis, previously known as “Ewing- like sarcomas” (ELS), and now distinguished from Ewing sarcoma in the recent WHO classification, 2020 (24). A multi-institutional European retrospective analysis conducted by Sparber-Sauer et al. focused on young patients (0– 24 years) with CIC-fused (n=31) and BCOR-rearranged (n=29) soft tissue sarcomas. They reported that despite local control achieved by surgery, patients experienced dismal outcomes due to disease progression or relapse. Three-year event-free survival were 44% and 41.2% for CIC and BCOR groups, respectively. Similarly, three- year overall survivals were 46.3% and 67.1% respectively. The study concluded that pediatric patients often present with large tumors and metastatic disease with dismal overall outcome and highlights the need for new treatment options, especially for CIC sarcomas, which often present with advanced and metastatic disease (37).

Interestingly, in our study, among the seven cases of sarcoma with BCOR genetic alterations, six patients (85.7%) exhibited high expression of various markers, five of them presented in advanced stage deemed unfit for upfront resection and four patients succumbed due to disease progression or relapse.

Regarding CIC-rearranged sarcoma, the outcomes observed in our four patients highlight the aggressive nature of this disease, with the three patients presenting with advanced stage succumbing to disease progression or relapse. Through the analysis of markers expression, two patient exhibited high expression of markers, one for VEGFR2 and the other for PDGFRα.

These findings highlight the necessity for further investigation into molecular angiogenic marker expression in these tumors, which are recognized for their poor prognosis. Such studies can help tailor treatment strategies to individual patients based on their specific molecular profiles, ultimately improving treatment outcomes in these challenging tumors. These findings also suggest that comprehensive studies involving larger cohorts of patients, both adult and pediatric) with the rarer BCOR genetic alterations or CIC-rearranged sarcoma are needed. Incorporating a broader range of tyrosine kinase markers are essential to validate and extend these initial findings and establish robust associations between marker expression profiles and clinical outcomes.

Understanding the expression patterns of angiogenic markers between different histological subtypes can provide valuable insights into the underlying mechanisms driving tumor progression, especially for those with poor prognosis, and may reveal potential therapeutic targets. This information could be crucial in predicting the response to multi-tyrosine kinase inhibitors such as pazopanib, which are now being incorporated into studies for advanced unresected cases of NRSTS. The integration of tyrosine kinase inhibitors (e.g. pazopanib, sorafenib) alongside chemotherapy regimens holds promise for augmenting local disease control and potentially enhancing treatment outcomes.

Nonetheless, the validation of these preliminary observations necessitates a comprehensive analysis involving a larger pediatric patient cohort. Such efforts will be indispensable for guiding future therapeutic strategies in the management of advanced stage sarcoma.

The major weakness of this study, which is common in pediatric sarcoma studies, is the heterogeneity of the sarcoma population. Even with a relatively large sample cohort with regard to pediatric high-grade NRSTSs, the numbers limit meaningful explorations based on histological subgroups, at least with respect to multivariate analysis.

In conclusion, we demonstrate that tyrosine kinase receptors are upregulated in sarcomas and show a distinct expression pattern in particular subgroups. High expression of VEGFR2 and PDGFRα significantly correlated with reduced patient survival in NRSTS and the identification of these protein signatures suggests that multi-tyrosine kinase inhibitor therapy might be a promising avenue to treat those patients. However, further investigations involving larger cohorts are warranted to explore the functional role of different markers and their impact on treatment outcome in different histological subtypes. This will be critical for informing the development of targeted therapeutic approaches tailored to the specific molecular characteristics of sarcomas, ultimately improving patient care and clinical outcomes.

## Data Availability

The original contributions presented in the study are included in the article/**Supplementary Material**. Further inquiries can be directed to the corresponding authors.

## Supplementary material

Supplementary Figure 1 high power field showing: **(A)** Negative expression for Vascular endothelial growth factor2 (VEGFR2). **(B)** Positive expression for VEGFR2. **(C)** Negative expression for c-Kit. **(D)** Positive expression for c-Kit.

Supplementary Figure 2 high power field showing: **(A)** Negative expression for platelet derived growth factor α (PDGFRα). **(B)** Positive expression for PDGFRα. **(C)** Negative expression for PDGFRβ. **(D)** Positive expression for PDGFRβ.
