# Supplementary material for: Patterns of expression of VEGFR2, PDGFRs and c-Kit in pediatric patients with high grade non-rhabdomyosarcoma soft tissue sarcoma
Source: Front Oncol. 2024 Oct 29;14:1480773. doi: 10.3389/fonc.2024.1480773 (PMC11555289; doi:10.3389/fonc.2024.1480773)
Supplement: Supplementary file 1 [file DataSheet1.docx]

**Supplementary table 1**: Immunoreactive score (IRS) method for VEGFR2, PDGFRα and PDGFRβ biomarkers:

| **Percent of positive cells x intensity of staining = Score (0-12)** | | | **IRS classification** |
| --- | --- | --- | --- |
| 0= negative  1= < 10% of positive cells  2= 10-50% of positive cells  3= 51-80% of positive cells  4= >80% of positive cells | 0= no color reaction  1= mild reaction  2= moderate reaction  3= intense reaction | 0-1= negative  2-3= mild  4-8= moderate  9-12= strongly positive | 0=negative  1= positive, weak expression  2= positive, intermediate expression  3= positive, strong expression |

**Abbreviations:** VEGFR2: vascular endothelial growth factor2, PDGFRα: platelet derived growth factorα, PDGFRβ: platelet derived growth factor β.

**Supplementary figures:**

**B**

**A**


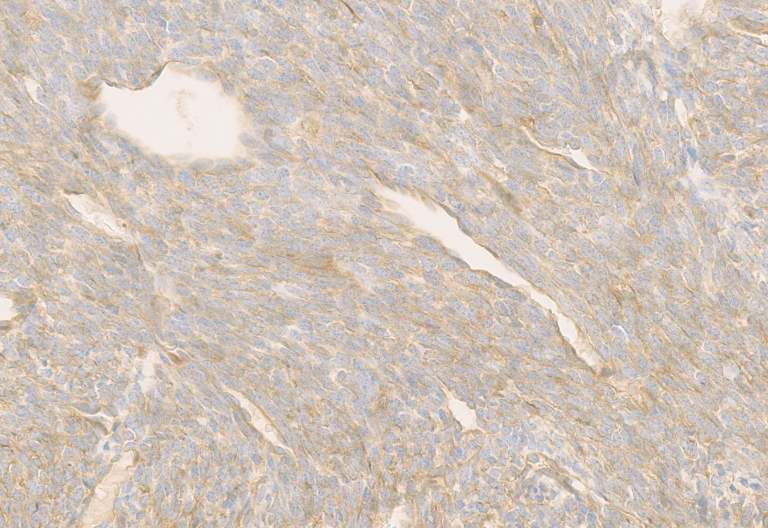

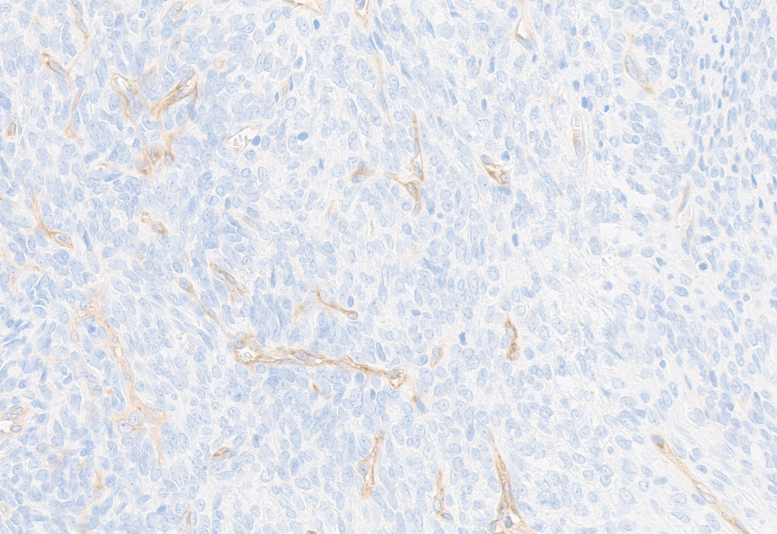


Positive VEGFR2

Negative VEGFR2

**D**

**C**


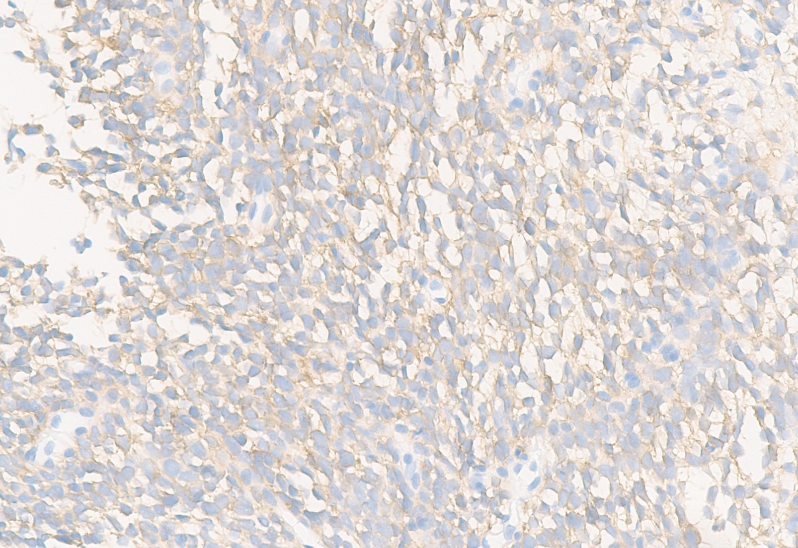

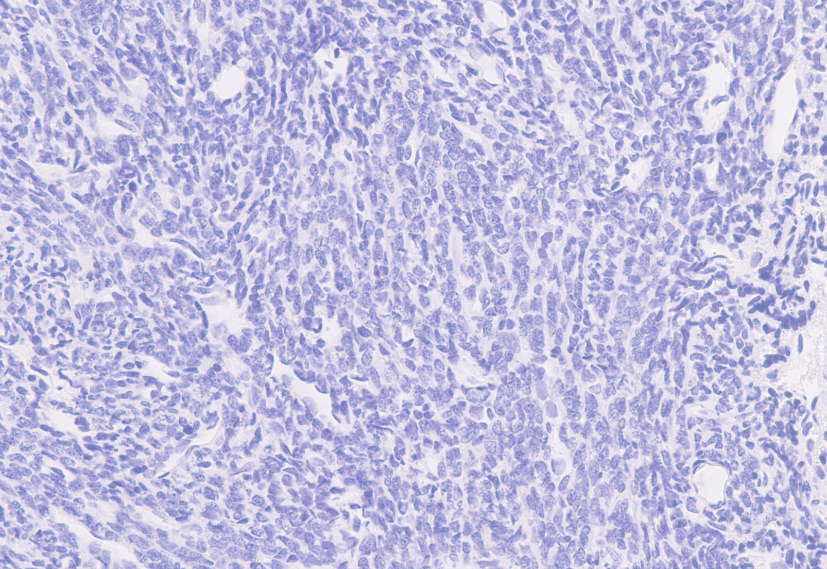


Positive c-Kit

Negative c-Kit

**Supplementary figure 1 (A, B, C, D)**

**B**

**A**


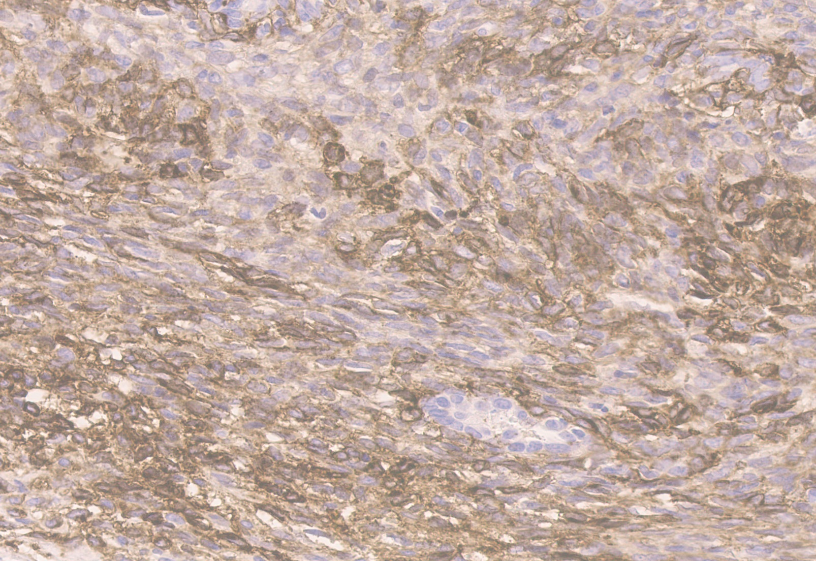

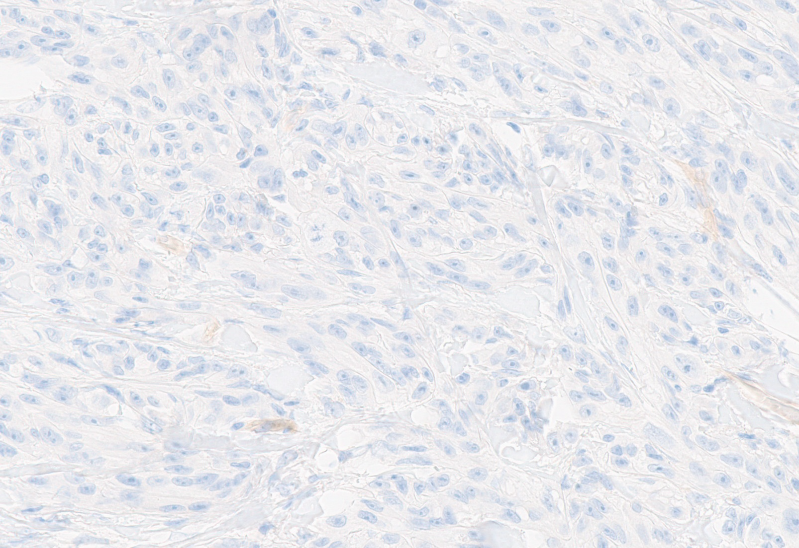


Negative PDGFRα

Positive PDGFRα

**D**

**C**


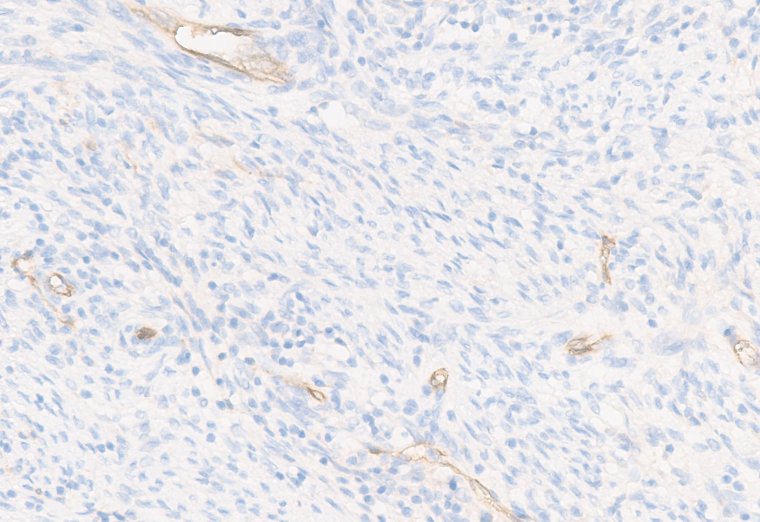

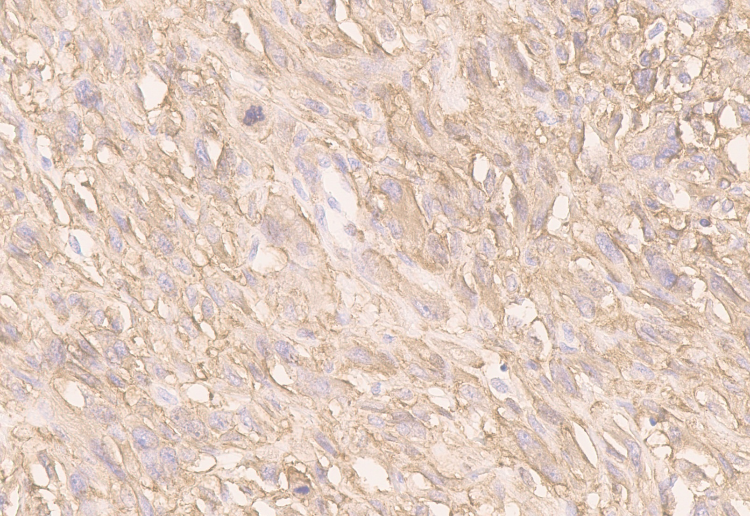


Positive PDGFRβ

Negative PDGFRβ

**Supplementary figure 2 (A, B, C, D)**
